# The Immediate Early Gene *Egr3* Is Required for Hippocampal Induction of *Bdnf* by Electroconvulsive Stimulation

**DOI:** 10.3389/fnbeh.2018.00092

**Published:** 2018-05-11

**Authors:** Kimberly T. Meyers, Ketan K. Marballi, Samuel J. Brunwasser, Briana Renda, Milad Charbel, Diano F. Marrone, Amelia L. Gallitano

**Affiliations:** ^1^Department of Basic Medical Sciences, College of Medicine Phoenix, University of Arizona, Phoenix, AZ, United States; ^2^Interdisciplinary Graduate Program in Neuroscience, Arizona State University, Tempe, AZ, United States; ^3^Medical Scientist Training Program, School of Medicine, Washington University in St. Louis, St. Louis, MO, United States; ^4^Department of Psychology, Wilfrid Laurier University, Waterloo, ON, Canada; ^5^Barrett, The Honors college, Arizona State University, Tempe, AZ, United States; ^6^Evelyn F. McKnight Brain Institute, The University of Arizona, Tucson, AZ, United States

**Keywords:** electroconvulsive therapy, immediate early genes, early growth response 3, brain-derived neurotrophic factor, schizophrenia, psychosis treatment

## Abstract

Early growth response 3 (*Egr3*) is an immediate early gene (IEG) that is regulated downstream of a cascade of genes associated with risk for psychiatric disorders, and dysfunction of *Egr3* itself has been implicated in schizophrenia, bipolar disorder, and depression. As an activity-dependent transcription factor, EGR3 is poised to regulate the neuronal expression of target genes in response to environmental events. In the current study, we sought to identify a downstream target of EGR3 with the goal of further elucidating genes in this biological pathway relevant for psychiatric illness risk. We used electroconvulsive stimulation (ECS) to induce high-level expression of IEGs in the brain, and conducted expression microarray to identify genes differentially regulated in the hippocampus of Egr3-deficient (-/-) mice compared to their wildtype (WT) littermates. Our results replicated previous work showing that ECS induces high-level expression of the brain-derived neurotrophic factor (*Bdnf*) in the hippocampus of WT mice. However, we found that this induction is absent in *Egr3*-/- mice. Quantitative real-time PCR (qRT-PCR) validated the microarray results (performed in males) and replicated the findings in two separate cohorts of female mice. Follow-up studies of activity-dependent *Bdnf* exons demonstrated that ECS-induced expression of both exons IV and VI requires *Egr3*. *In situ* hybridization demonstrated high-level cellular expression of *Bdnf* in the hippocampal dentate gyrus following ECS in WT, but not *Egr3*-/-, mice. *Bdnf* promoter analysis revealed eight putative EGR3 binding sites in the *Bdnf* promoter, suggesting a mechanism through which EGR3 may directly regulate *Bdnf* gene expression. These findings do not appear to result from a defect in the development of hippocampal neurons in *Egr3*-/- mice, as cell counts in tissue sections stained with anti-NeuN antibodies, a neuron-specific marker, did not differ between *Egr3*-/- and WT mice. In addition, Sholl analysis and counts of dendritic spines in golgi-stained hippocampal sections revealed no difference in dendritic morphology or synaptic spine density in *Egr3*-/-, compared to WT, mice. These findings indicate that *Egr3* is required for ECS-induced expression of *Bdnf* in the hippocampus and suggest that *Bdnf* may be a downstream gene in our previously identified biologically pathway for psychiatric illness susceptibility.

## Introduction

The risk to develop neuropsychiatric illnesses is determined by both genetic and environmental factors. We have hypothesized that immediate early gene (IEG) transcription factors are poised to modulate this dual contribution, as they are rapidly activated in the brain in response to environmental stimuli and, in turn, influence numerous neurobiological processes that are dysfunctional in the brains of patients with mental illness (Gallitano-Mendel et al., 2007, 2008; Huentelman et al., 2015). These include memory formation and synaptic plasticity (Gallitano-Mendel et al., 2007; Forbes et al., 2009), myelination (Jessen and Mirsky, 2002; Davis et al., 2003; Dwork et al., 2007), vascularization (Mechtcheriakova et al., 1999; Fahmy et al., 2003; Hanson and Gottesman, 2005), and growth factor response (Moises et al., 2002; Fahmy and Khachigian, 2007; Zakharyan et al., 2014). Our prior work has focused on investigating the functions of the IEG transcription factor early growth response 3 (*Egr3*), as it is regulated downstream of numerous proteins that are associated with risk for neuropsychiatric disorders (Gallitano-Mendel et al., 2007, 2008; Huentelman et al., 2015). We have previously reported that *Egr3*-deficient (-/-) mice have behavioral abnormalities consistent with animal models of mental illness, including schizophrenia and bipolar disorder, that are rescued by the antipsychotic medications used to treat these disorders (Gallitano-Mendel et al., 2007, 2008). *EGR3* has been associated with risk for schizophrenia in Japanese, Korean, Han Chinese, and US populations of European descent (Yamada et al., 2007; Kim et al., 2010; Zhang et al., 2012; Huentelman et al., 2015) and levels of *EGR3* are reduced in the brains of schizophrenia patients (Mexal et al., 2005; Yamada et al., 2007). Recently, *EGR3* was identified as a master regulator in a network of genes differentially expressed in the postmortem brains of bipolar disorder patients, compared with controls, in two independent cohorts (Pfaffenseller et al., 2016). In addition, although *EGR3* was not identified within one of the 108 loci found to be associated with schizophrenia risk in the Psychiatric Genomics Consortium genome-wide association study (GWAS), numerous genes encoding proteins that regulate or are regulated by *EGR3* do map to these schizophrenia-associated loci (Schizophrenia Working Group of the Psychiatric Genomics Consortium, 2014; Marballi and Gallitano, 2018).

The few confirmed downstream target genes of EGR3 have also been implicated in neuropsychiatric disorders. These include glutamic acid decarboxylase A4 (*GABRA4*; Roberts et al., 2005, 2006), and activity-regulated cytoskeleton associated protein (*Arc-Arg3.1*; Li et al., 2005). In addition, our prior studies identified a specific deficit in GluN2B-containing *N*-methyl D-aspartate receptors (NMDARs) in the hippocampus of *Egr3*-deficient (-/-) mice, indicating a requirement of *Egr3* for the normal function of this receptor (Gallitano-Mendel et al., 2007). Hypofunction of NMDARs is one of leading hypotheses for the etiology of schizophrenia (Javitt and Zukin, 1991; Olney et al., 1999). Indeed, GRIN2A, the gene encoding the NMDAR subunit GluN2A, is also located at one of the 108 schizophrenia-associated loci (Schizophrenia Working Group of the Psychiatric Genomics Consortium, 2014).

Based on these prior studies, we hypothesized that other EGR3 target genes in the brain may be critical contributors to neuropsychiatric disorders. Since neuronal expression of IEGs is activity dependent, we used electroconvulsive seizure (ECS) to maximally activate IEGs, and compared hippocampal gene expression between mice lacking *Egr3* and their wildtype (WT) littermates. This experimental approach is particularly relevant as ECS is an experimental model of electroconvulsive therapy (ECT), which remains one of the most effective treatments for severe mood and psychotic disorders. However, the mechanisms underlying the efficacy of ECT remain elusive.

Here we show that induction of brain-derived neurotrophic factor (*Bdnf*), a critical neurotrophin involved in a wide range of neurobiological functions, is significantly diminished or absent in the hippocampus of mice lacking *Egr3*. Dysfunction of BDNF has been implicated in numerous neuropsychiatric disorders (Autry and Monteggia, 2012), including bipolar disorder and schizophrenia (Durany et al., 2001). Like *Egr3, Bdnf* is also induced in the hippocampus following ECS, and this expression is associated with the effectiveness of antidepressant treatments in reversing mood disorder-like phenotypes in rodents (Altar et al., 2003; Adachi et al., 2008; Inta et al., 2013).

## Materials and Methods

### Mice

Previously generated *Egr3*-/- mice (Tourtellotte and Milbrandt, 1998) were backcrossed to C57BL/6 mice for greater than 20 generations. Studies were conducted on homozygous adult littermate progeny of heterozygote matings, and assigned as “matched pairs” at the time of weaning. Matched pairs were exposed to identical conditions for all studies. The term WT refers to +/+ littermates of *Egr3*-/- mice generated from these crossings. Male mice ages 6–12 months (*n* = 4) were utilized for the microarray and follow-up quantitative real-time PCR (qRT-PCR) study. Results were validated in two separate cohorts of female *Egr3* mice. The first cohort included older adult females, ages 12–15 months, from the same C57BL/6 background (*n* = 4–5 per group). A second cohort of females, age 3.5–6 months (*n* = 4–5 per group), had a mixed background resulting from crossing the above *Egr3* C57BL/6 background mice to a GENSAT reporter line in a mixed FVB/N and Swiss Webster background. Since these animals contain a bacterial artificial chromosome expressing EGFP, in addition to a mixed background, results were analyzed separately from other groups.

Animals were housed on a 12 h light/dark schedule with *ad libitum* access to food and water. All studies were performed in accordance with the University of Arizona, Institutional Animal Care and Use Committee (IACUC). This study was carried out in accordance with the recommendations of IACUC guidelines, IACUC. The protocol was approved by IACUC.

### Electroconvulsive Stimulation and Tissue Collection

To anesthetize the corneas of all animals, 0.5% proparacaine hydrochloride ophthalmic solution (Akorn, Inc., Lake Forest, IL, United States) was applied 5 min prior to electroconvulsive stimulation (ECS). Male mice utilized in the microarray study underwent ECS without general anesthesia. Female mice used in the replication studies were anesthetized with isoflurane (VetOne, Boise, ID, United States) administered in an enclosed chamber at a flow rate of 0.5 mL/min in oxygen. Animals were removed from the chamber after 2 min of full anesthetization, transferred to room air to recover to a level of light anesthesia, and then administered electrical stimulation of 260 A for 1 ms duration and a pulsewidth of 0.3 mm, 1 ms. (Ugo Basile, Varese, Italy) via orbital electrodes. Mice were observed to undergo seizure, and were placed in their home cage to recover for one hour prior to sacrifice. Control animals remained in their home cages undisturbed.

### Tissue Collection and RNA Isolation

Animals were sacrificed via isoflurane overdose, followed by decapitation. The brains were removed, rinsed in sterile ice-cold phosphate buffered saline (PBS), and hemisected along the central sulcus into right and left hemispheres for further studies to quantify both the expression of mRNA with qRT-PCR and *in situ* hybridization, respectively.

Hippocampal tissue was dissected and immediately placed in RNAlater (Ambion, Waltham, MA, United States). Tissue was transferred to 1.5-mL Eppendorf tubes, immediately placed on dry ice, and transferred to -80°C until further use. For male cohort microarray and follow-up qRT-PCR, RNA was isolated using TRIzol reagent (Life Technologies, Carlsbad, CA, United States) followed by phenol/chloroform extraction, chloroform extraction, and isopropanol precipitation, per the manufacturer’s protocol. RNA was resuspended in RNAse-free water and quantitated by spectrophotometry. RNA quality and concentration was validated by Agilent Bioanalyzer 2100 prior to microarray analysis and reverse transcription for qRT-PCR. An aliquot of the RNA samples was sent to the Microarray Resource Center, Yale/NIH Neuroscience Microarray Center (New Haven, CT, United States) for analysis using an Illumina Mouse WG6 v3.0 expression beadchip microarray. For female cohorts, RNA isolation was performed using TRI reagent (Sigma-Aldrich, St. Louis, MO, United States) and MagMax^TM^ Total RNA isolation kit (Ambion, Waltham, MA, United States) according to the manufacturer’s protocol, and quantified using the NanoDrop ND-1000 spectrophotometer (Thermo Scientific, Waltham, MA, United States).

### Microarray Procedure and Analysis

Data analysis and quality control was initially performed using Gene Pattern (Reich et al., 2006), with normalization using the cubic spline method using the following settings (FDR < 0.05) to determine significantly differentially expressed genes between the WT and *Egr3*-/- groups 1 h following ECS. This was the timepoint at which we expected the most enrichment for EGR3 transcription factor targets, as *Egr3* is maximally induced by ECS (O’Donovan et al., 1998). A parallel analysis using the Illumina Genome studio 2010 software was used to determine differentially expressed genes by comparing the same two groups using the following settings: background subtraction, quantile normalization, *p* < 0.05 (*T*-test). Finally, a list of common differentially expressed genes using both programs was generated. Complete microarray results will be published separately.

### qRT-PCR

For qRT-PCR studies, mRNA was reverse transcribed into cDNA using a standard protocol (Maple et al., 2015), and used as a template for qRT-PCR using FastStart SYBR Green Master mix (Roche Applied Science, Indianapolis, IN, United States) on a 7500 Fast Real-Time PCR machine (ThermoFisher Scientific, Waltham, MA, United States). Each sample was amplified in triplicate for both *Bdnf* and the housekeeping gene phosphoglycerate kinase 1 (*Pgk1*). *Pgk1* was selected for use as a housekeeping gene as one of the least changed genes across experimental groups in the microarray data and validated by qRT-PCR in all cohorts.

General *Bdnf* primers were used to assess overall levels of total *Bdnf* mRNA (*Bdnf* F, R) (Tallaksen-Greene et al., 2014). These primers target a region common to all 12 transcripts of *Bdnf* as revealed by NCBI primer blast analysis. For follow-up exon-specific studies, we focused on *Bdnf* exons IV and VI as removal of these exons *in vivo* significantly reduces BDNF protein levels in the mouse hippocampus (Maynard et al., 2016). In addition, *Bdnf* transcripts containing exon IV and VI are highly induced *in vivo* following ECS in the mouse frontal cortex (Martinowich et al., 2011). Exon specific primers for *Bdnf* exons IV and VI (Zheng and Wang, 2009) were used in our study. Fold changes in gene expression and data were plotted using the 2^-ΔCT^ method (Le et al., 2013). Primer sequences used were as follows, *Bdnf* F: TGG CCC TGC GGA GGC TAA GT; *Bdnf* R: AGG GTG CTT CCG AGC CTT CCT; *Pgk1* F: TGT TAG CGC AAG ATT CAG CTA GTG; *Pgk1* R: CAG ACA AAT CCT GAT GCA GTA AAG AC; *Bdnf* exon IV F: CTC CGC CAT GCA ATT TCC AC; *Bdnf* exon VI F: GTG ACA ACA ATG TGA CTC CAC; and *Bdnf* exon IV/VI R: GCC TTC ATG CAA CCG AAG TA.

### Prediction of EGR3 Binding Sites in *Bdnf* Gene Regulatory Regions

Bioinformatic identification of putative EGR binding sites in the promoter of the mouse *Bdnf* gene was carried out using the TFBIND^1^ (Tsunoda and Takagi, 1999) website that utilizes the transcription factor database TRANSFAC R. 3.3. Briefly, the nucleotide sequence 4000 bp upstream of the start ATG in the mouse *Bdnf* gene was exported from the UCSC genome browser^2^ using genome assembly GRCm38/mm10 and used to query the TFBIND site. This generated an output that showed all the putative binding sites for the EGR3 TF.

### Golgi-Cox Preparation

Tissue was treated using the Golgi-Cox method, as previously described (Gibb and Kolb, 1998; Gallitano et al., 2016). Briefly, 10 animals (5 *Egr3*-/- and 5 WT) were decapitated under isoflurane and the brains were rapidly extracted. The brains were rinsed in 0.9% saline and immersed in Golgi-Cox solution for at least 14 days followed by 30% sucrose for at least 3 days. The solution was made in the laboratory from ingredients purchased from Sigma-Aldrich (St. Louis, MO, United States). Brains were then sectioned on a Vibratome at 200 μm and mounted on gelatin-coated slides.

### Golgi-Cox Imaging and Analysis

Ten fully impregnated cells were selected in each region, from the hippocampal Cornu Ammonis (CA) regions CA1 and CA3, and the dentate gyrus (DG) of each animal, and were imaged in a z-stack throughout the thickness of the section using a brightfield microscope equipped with a digital camera (AmScope, Irvine, CA, United States). Using ImageJ (NIH) or Metamorph (Molecular Devices) software, several measures of dendritic arborization were obtained (**Figure 3**). The Sholl technique (Sholl, 1953) involves overlaying the neuron with a series of concentric circles (20 μm apart) and recording the number of dendritic processes intersecting each circle to a maximal distance of 380 μm. In addition, branching order was calculated for all cells. The primary dendrites originating from the soma are assigned a branch order of one, while dendritic processes originating from that dendrite are second-order branches, and each subsequent bifurcation is assigned a progressively higher branch order (van Pelt et al., 1986). Dendritic spine density and morphology were analyzed at 100x under oil immersion. Dendritic spines were counted in a sample of two random 20-μm-long segments in several distinct regions of each cell according to the classification of Lorente de No (1934). For CA1 and CA3, these regions were: the basal tuft (stratum oriens), the mid-point of the apical tuft (near the distal edge of the stratum radiatum), and the distal tip of the apical tuft (stratum lacunosum moleculare). In the DG, dendritic spines were counted in the inner molecular layer (IML), middle molecular layer (MML), and outer molecular layer (OML) as defined by dividing the length of the molecular layer into three equal parts.

### Immunohistochemistry

Animals were sacrificed by isoflurane euthanasia and were perfused with PBS followed by fixation with 4% paraformaldehyde (PFA). Brains were harvested and were post-fixed in 4% PFA for 24 h, rinsed in Tris-buffered saline (TBS, pH 7.4), transferred into 30% sucrose, and stored at 4°C until saturated, rinsed, and stored in TBS at 4°C. Tissue was sectioned coronally at a thickness of 20 μm on a sliding microtome and stored until further processing in a cryopreservative solution.

#### Immunohistochemistry

Tissue was rinsed in TBS, 3x × 5 min. Endogenous peroxidase was quenched with 1% H_2_O_2_ × 10 min in a buffer/detergent solution (0.4% Triton-X in TBS). The reaction was stopped by rinsing the tissue 3x × 5 min in TBS. Tissue was blocked with 4% normal goat serum (Sigma-Aldrich, St. Louis, MO, United States) prepared in 0.4% Triton-X in TBS at room temperature (RT) for 2 h. Polyclonal rabbit primary antibody raised against neuronal nuclei, NeuN (1:1000, Millipore, Cat #ABN78) was prepared in 4% normal goat serum in 0.4% Triton-X in TBS and was incubated at RT for 1 h with a 24-h incubation at 4°C. A secondary biotinylated antibody raised against rabbit (1:1000, Vector Labs, #BA-1000) was prepared in 4% normal goat serum in 0.4% TBS-TX and was incubated with the tissue for 1 h at RT. Avidin–biotin complex (ABC; ThermoFisher Scientific, Waltham, MA, United States) was prepared at a concentration of 1:1000 in TBS. Tissue was incubated in ABC for 1 h at RT. Detection of the antigen was performed with 3,3′-diaminobenzidine (DAB) kit (ImmPACT DAB Peroxidase Substrate, Cat No. SK-4105, Vector Laboratories, Burlingame, CA, United States). Tissue sections were imaged with a Zeiss Imager M2 microscope using a 40x objective, photographed using an Axiocam 506 camera, and tiled together using Zen 2012 software (Zeiss Microscopy, Oberkochen, Germany).

Neurons were counted from defined anatomical regions of CA1 and CA3 pyramidal neurons, as well as both the suprapyramidal (DG_sp_) and infrapyramidal (DG_ip_) blades of the DG. For each brain, neurons were counted from four sections spanning a total rostral–caudal distance of 200 μm including, and caudal to, coordinates: interaural, 1.74 mm; bregma, 2.06 mm (Paxinos and Franklin, 2001). The number of neurons within each analyzed region of the hippocampus was estimated using Abercrombie’s (1946) correction.

### *In Situ* Hybridization

#### Tissue Preparation

Tissue was flash frozen in -40°C methylbutane and was sectioned coronally at 10 μm thickness with a cryostat (Leika, Buffalo Grove, IL, United States), mounted on super-charged slides and stored at -80°C. Tissue sections from matched experimental pairs of animals were mounted on a single slide to control for possible staining differences (*Egr3*-/- control, *Egr3*-/- ECS, WT control, and WT ECS).

#### Probe Generation

Mouse BDNF DNA plasmid (accession #X55573) was kindly donated by Dr. Stanley Watson, University of Michigan, Ann Arbor. The DNA sequence was validated by comparison to *Bdnf* reference sequence using FinchTV software (Geospiza, Inc., Seattle, WA, United States). Sense and antisense strands were generated by *in vitro* transcription with the AMBION Maxiscript T7 kit (Ambion, Waltham, MA, United States), labeled with digoxigenin RNA labeling mix (Roche, Basel, Switzerland), and purified with mini columns according to the manufacturer’s protocol. (Roche, Basel, Switzerland).

#### *In Situ* Hybridization

Tissue was fixed in 4% PFA at 4°C for 5 min., followed by a wash in 2x SSC for 2 min at RT. Tissue was acetylated in acetic anhydride (0.625%) for 10 min at RT, rinsed in sterilized water, and delipidized with a 1:1 mixture of acetone and methanol for 5 min. Tissue was rinsed in 2x SSC for 5 min, before application of the probe (300 ng) prepared in hybridization buffer (Sigma, St. Louis, MO, United States). The probe was applied directly to the mounted tissue (250 μL/slide) and was cover-slipped (FisherBrand, Fisher Scientific). Tissue was hybridized in a humid, sealed chamber with a mixture of 2x SSC and formamide overnight at 56°C. Stringency washes were performed on Day 2 to remove any non-specific binding (protocol available on request). Endogenous peroxidase was quenched with 2% hydrogen peroxide, and tissue was permeabilized with 1x SSC-Tween followed by rinsing in 0.1 M TBS (pH 7.4). The tissue was blocked with a mixture of 0.5% TSA blocking buffer (PerkinElmer, Waltham, MA, United States) and 5% normal sheep serum prepared in 0.1 M TBS. Anti-digoxigenin (Roche, Basel, Switzerland; 1:400 dilution in blocking buffer) was applied to the slides at RT for 2 h. Slides were washed in 0.1 M TBS-Tween 4x × 15 min at RT and the antibody was detected through alkaline phosphatase development, SIGMA*FAST* BCIP/NBT tablets (Sigma, St. Louis, MO, United States). Slides were imaged with bright-field microscopy (Axiovision, Zeiss, Oberkochen, Germany) at a magnification of 40x.

### Statistical Analysis

All statistical analyses for qRT-PCR and *in situ* data were carried out using graphpad prism. In all cases, two-way analysis of variance (ANOVA) was performed followed by Tukey’s *post hoc* test, corrected for multiple comparisons, and significance was set at *p* < 0.05. Golgi-Cox impregnated granule cells and pyramidal cells were analyzed in independent ANOVAs. Sholl data were analyzed by repeated-measures ANOVA, while branching order and spine density measures used general factorial ANOVA, with genotype and hippocampal region (i.e., either CA1, CA3a/b, and CA3c or DGsp and DGip) as factors. The neuron density (number of neurons per unit area) was compared between WT and *Egr3*-/- brains using a 2 × 5 ANOVA with genotype and hippocampal region (i.e., CA1, CA3a/b, CA3c, DG_sp_, and DG_ip_) as factors.

## Results

### Activity-Dependent Hippocampal *Bdnf* Expression Depends on *Egr3*

To identify genes regulated by *Egr3* in the mouse hippocampus, we compared the complement of genes expressed in *Egr3*-/- mice to that of WT mice. Since *Egr3* is an activity-dependent IEG, and thus expressed at low levels in the absence of a stimulus, we used ECS to maximally activate IEG expression in both *Egr3*-/- and WT mice, and compared levels of induced genes to those at baseline. We conducted an expression microarray to screen for genes that were differentially expressed in response to seizure in the hippocampus of male WT, compared to *Egr3*-/-, mice.

Results of the microarray showed 65 genes to be differentially expressed between WT and *Egr3*-/- mice following ECS. Of these, 40 genes were increased (greater than 1.5-fold), while 13 were decreased, in WT mice following ECS compared to *Egr3*-/- mice following ECS. Twelve genes were minimally changed across both groups (1–1.4-fold). *Bdnf* was the only growth factor among these 65 putative EGR3-dependent, ECS-induced genes. Complete results of the microarray will be published separately.

**FIGURE 1:**
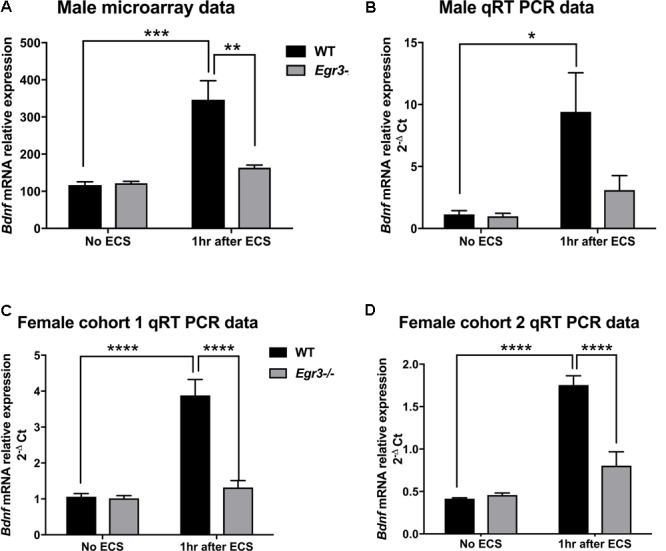
Electroconvulsive seizure (ECS)-induced hippocampal *Bdnf* expression is *Egr3*-dependent. Expression microarray analysis **(A)**, and validation using qRT-PCR **(B–D)**, of hippocampal *Bdnf* expression from WT, and *Egr3*–/–, mice at baseline (no ECS) and 1 h after ECS. Two-way ANOVAs showed significant interaction of genotype and ECS treatment on *Bdnf* expression in **(A)** male microarray data (*p* = 0.004) and qRT-PCR data in **(C)** female cohort 1 (*p* = 0.0005) and **(D)** female cohort 2 (*p* = 0.0003), and **(B)** significant effect of treatment in original male cohort (*p* = 0.0098; *n* = 4–5 animals/group; ^∗^*p* < 0.05, ^∗∗^*p* < 0.01, ^∗∗∗^*p* < 0.001, and ^∗∗∗∗^*p* < 0.0001 controlled for multiple comparisons).

*Bdnf* was of particular interest to us, as it has been shown to contribute to the therapeutic effects of antidepressant treatments, including ECS, in rodent models (Altar et al., 2003; Adachi et al., 2008; Inta et al., 2013). Results of the expression microarray demonstrated that, in WT mice, ECS produced a 2.5-fold increase in *Bdnf* mRNA levels, measured 1 h following seizure, compared to baseline unstimulated controls. This induction is dependent upon *Egr3*, as *Egr3*-/- mice did not show a change in *Bdnf* mRNA expression following seizure (**Figure 1A**). A two-way ANOVA revealed a significant interaction of genotype and treatment [*F*_(1,12)_ = 12.65, *p* = 0.004]. *Post hoc* analysis showed that WT mice had significantly higher levels of *Bdnf* induction after ECS compared to the untreated WT (*p* < 0.001) mice, while *Egr3*-/- mice did not show a significant increase in *Bdnf* expression following ECS compared to baseline. In addition, there was a significant difference in *Bdnf* levels between WT and *Egr3*-/- mice following ECS (*p* < 0.01).

To validate these findings, we performed qRT-PCR from the RNA samples that were used for the expression microarray (**Figure 1B**). ECS induced a greater than six-fold increase in *Bdnf* expression in WT mice (compared to non-stimulated control), while it failed to induce a significant increase in *Bdnf* expression in the *Egr3*-/- mice. These results validate the microarray data. A main effect of treatment was observed using two-way ANOVA [*F*_(1,12)_ = 9.393, *p* = 0.0098] and *post hoc* analysis demonstrated that ECS-treated WT mice had significantly higher levels of *Bdnf* after ECS compared to the untreated WT (*p* < 0.05). ECS did not result in a significant change in *Bdnf* expression in *Egr3*-/- mice (*p* = 0.8133).

To determine whether the requirement of *Egr3* for activity-dependent induction of *Bdnf* is sex specific, we conducted ECS in female *Egr3*-/- and WT mice. **Figure 1C** shows that, as in male mice, ECS induced a significant increase (3.8-fold) in *Bdnf* mRNA in WT female mice, but did not result in a statistically significant increase in *Egr3*-/- mice. Cohort 1 (**Figure 1C**) showed a significant interaction of genotype and treatment [*F*_(1,14)_ = 20.52, *p* = 0.0005] and *post hoc* analysis revealed significant elevations in *Bdnf* levels post ECS in the WT group compared to WT controls (*p* < 0.0001) and *Egr3*-/- ECS-treated mice (*p* < 0.0001), respectively.

To determine whether the effect was also seen in younger animals, we used a second cohort of female mice. **Figure 1D** shows a similar result to that seen in adult male, and older female, C57BL/6 mice, that ECS produces a fourfold increase in *Bdnf* expression in WT mice that is dependent upon *Egr3*. Two-way ANOVA demonstrated a significant interaction of genotype and treatment [*F*_(1,13)_ = 23.85, *p* = 0.0003]. *Post hoc* analysis revealed significant elevations in *Bdnf* post ECS in the WT group compared to WT controls (*p* < 0.0001) and to *Egr3*-/- ECS-treated mice (*p* < 0.0001). No significant effect of ECS on *Bdnf* induction was observed in *Egr3*-/- mice in either group. These data suggest that *Bdnf* induction by ECS is dependent, at least in part, on *Egr3*.

### ECS-Induced Exon IV and Exon VI *Bdnf* Expression Is *Egr3* Dependent

Given that the *Bdnf* gene has multiple splice variants, we next examined whether *Egr3* is required for ECS-induced expression of specific splice variants. This was of particular interest as ECS induces specific exon variants in the mouse brain *in vivo* (Martinowich et al., 2011). Martinowich et al. (2011) showed that mRNAs containing exon IV and exon VI are highly upregulated following ECS compared to untreated mice, and mice that lack exon IV fail to show induction of six splice variants following ECS *in vivo* (Martinowich et al., 2011). In addition, both exon IV and exon VI containing mRNAs maximally contribute to total BDNF protein levels in the mouse hippocampus (Maynard et al., 2016). Since exons IV and VI are required for expression of the remaining BDNF exons, we decided to evaluate levels of transcripts containing these two critical exons in WT and *Egr3*-/- mice following ECS compared with untreated controls.

**FIGURE 2:**
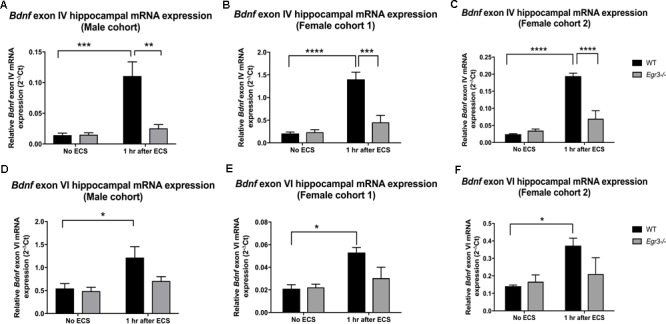
Electroconvulsive seizure (ECS)-mediated hippocampal induction of *Bdnf* exon IV and VI requires *Egr3*. Exon IV **(A–C)** and VI **(D–F)** hippocampal *Bdnf* expression determined using qRT PCR from WT, and *Egr3*–/–, mice at baseline (no ECS) and 1 h after ECS. Two-way ANOVAs showed significant interactions of genotype and ECS treatment on *Bdnf* exon IV expression in **(A)** original male cohort data (*p* = 0.0041), **(B)** female cohort 1 (*p* = 0.0019), and **(C)** female cohort 2 (*p* = 0.0001). Two-way ANOVAs showed significant effect of treatment on *Bdnf* exon VI expression in original **(D)** male cohort (*p* = 0.0099), **(E)** female cohort 1 (*p* = 0.0064), and **(F)** female cohort 2 (*p* = 0.0237; *n* = 4–5 animals/group; ^∗^*p* < 0.05, ^∗∗^*p* < 0.01, ^∗∗∗^*p* < 0.001, and ^∗∗∗∗^*p* < 0.0001 controlled for multiple comparisons).

Quantitative real-time PCR using exon specific primers showed that WT mice demonstrated a 10-fold increase in *Bdnf* exon IV expression, 1 h post-ECS that was absent in *Egr3*-/- mice (**Figure 2A**). Two-way ANOVA revealed a significant interaction of genotype and treatment [*F*_(1,12)_ = 12.52, *p* = 0.0041]. *Post hoc* analysis indicated that WT mice had significantly higher levels of *Bdnf* exon IV mRNA induction after ECS compared to the untreated WT (*p* < 0.001) mice. *Egr3*-/- mice did not show a significant increase in *Bdnf* exon IV expression following ECS compared to baseline, but showed decreased *Bdnf* exon IV expression compared to WT mice following ECS (*p* < 0.01). Similar results were seen in both female cohorts. Female cohort 1 (**Figure 2B**) WT mice exhibited a sevenfold induction in *Bdnf* exon IV post ECS compared to baseline. Two-way ANOVA showed a significant interaction of genotype and treatment [*F*_(1,14)_ = 14.46, *p* = 0.0019]. *Post hoc* analysis demonstrated that WT mice had a significantly greater induction of *Bdnf* exon IV following ECS compared to baseline (*p* < 0.0001) and compared to *Egr3*-/- mice post ECS (*p* < 0.001). WT mice from the second female cohort demonstrated a ninefold induction in *Bdnf* exon IV post ECS compared to non-stimulated controls (**Figure 2C**). Two-way ANOVA revealed a significant interaction of genotype and treatment [*F*_(1,13)_ = 30.24, *p* = 0.0001]. *Post hoc* analysis revealed WT mice showed a significant induction of *Bdnf* exon IV post ECS compared to baseline (*p* < 0.0001) and *Egr3*-/- mice that underwent ECS (*p* < 0.0001).

In comparison, *Bdnf* exon VI mRNA was induced 2.2-fold in male WT mice following ECS compared to baseline that was absent in *Egr3*-/- mice (**Figure 2D**). Two-way ANOVA showed a significant effect of treatment [*F*_(1,12)_ = 9.361, *p* = 0.0099] and *post hoc* analysis revealed a significant induction of *Bdnf* exon VI in WT mice that underwent ECS vs. non-stimulated WT mice (*p* < 0.05). These data were replicated in both female cohorts. WT mice showed a 2.24- (**Figure 2E**) and a 2.64-fold (**Figure 2F**) induction post ECS compared to baseline, in the first and second female cohorts, respectively, that was absent in *Egr3*-/- mice. Two-way ANOVA revealed a significant effect of treatment in both female cohorts; female cohort 1 [*F*_(1,14)_ = 10.26, *p* = 0.0064] and female cohort 2 [*F*_(1,13)_ = 6.557, *p* = 0.0237]. *Post hoc* analysis revealed that significant induction of *Bdnf* exon VI in WT mice that underwent ECS compared to baseline (*p* < 0.05) in both female cohorts, while this effect was not observed in *Egr3*-/- mice.

### ECS-Induced *Bdnf* Expression in Dentate Gyrus Requires *Egr3*

**FIGURE 3:**
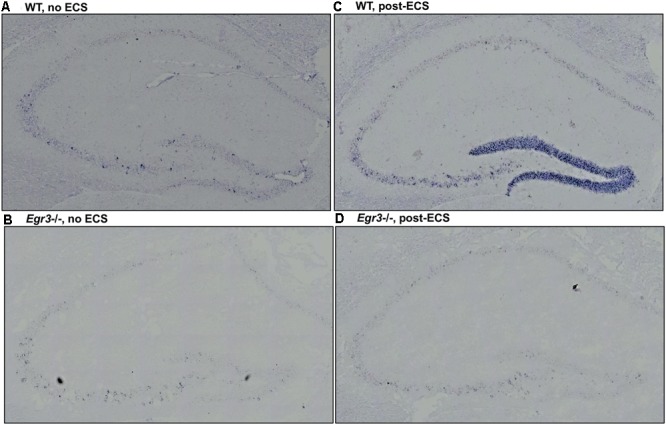
*Bdnf* induction in the hippocampal dentate gyrus requires *Egr3*. Representative tissue sections demonstrating *in situ* hybridization labeling of *Bdnf* mRNA expressing cells in the hippocampus of WT and *Egr3*–/– mice at baseline **(A,B)** and 1 h following ECS **(C,D)**. **(A)** *Bdnf* is expressed sparsely in CA3, CA2, and the DG of WT mice at baseline. **(C)** ECS induces high-level *Bdnf* expression in the DG, and increased expression in CA3, CA2, and CA1 of WT mice. **(B)** *Bdnf* expression in *Egr3*–/– mice is limited to rare cells in the CA3 region of the hippocampus, with no clear expression above background in the DG, CA2, or CA1, at baseline. **(D)** ECS produces little to no increase in *Bdnf* expression in the DG, CA3, CA2, and CA1 regions of *Egr3*–/– mice.

To identify the location of *Bdnf* expression in the hippocampus following ECS, and to determine in which regions it is dependent on EGR3, we conducted *in situ* hybridization to detect expression of *Bdnf* mRNA in hippocampal tissue sections. *In situ* hybridization histochemistry was performed on tissue sections from female *Egr3*-/- and WT mice at baseline, and 1-h post ECS. In WT mice, ECS resulted in a strong induction of *Bdnf* mRNA in the DG of the hippocampus 1 h after ECS (**Figure 3C**) compared with baseline, unstimulated controls (**Figure 3A**). Expression of *Bdnf* in the hippocampus of WT mice at baseline is found in sparsely distributed individual cells in the DG as well as CA regions 2 and 3 (**Figure 3A**). One hour following ECS, high level *Bdnf* expression is evident uniformly throughout the dorsal and ventral blades of the DG (**Figure 3C**). There is no evident increase in the individual cellular pattern of labeling in CA2 and CA3 (**Figure 3C**). In *Egr3*-/- mice ECS did not result in increased expression of *Bdnf* (**Figure 3D**) compared to baseline (**Figure 3B**), and neither condition demonstrated strong labeling of *Bdnf* in the DG, or in CA1, CA2, or CA3 regions (**Figures 3B,D**).

### EGR3 Binding Sites Are Present in *Bdnf* Regulatory Regions

**FIGURE 4:**
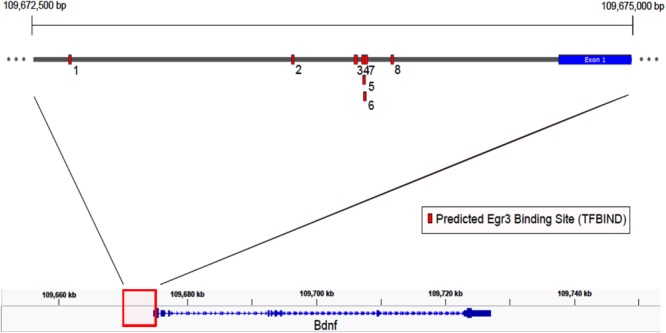
Map of predicted EGR3 binding sites in *Bdnf* upstream region. EGR3 binding sites identified in **Table 1** are indicated in red. Positions are shown relative to exon 1 of the mouse *Bdnf* gene as identified in the NCBI Refseq database. Numbers under each binding site correspond to numbered rows in **Table 1**.

If *Egr3* is required to directly regulate expression of *Bdnf*, then EGR3 binding sites should be present in the promoter of the *Bdnf* gene. The genomic sequence 4000 bp upstream of the *Bdnf* transcription start site contains eight high-probability putative binding sites for EGR3 (**Figure 4** and **Table 1**), with a cutoff value of 0.74 designated by TFBIND that calculates transcription factor-specific cutoffs using an algorithm (Tsunoda and Takagi, 1999). These sites represent a potential mechanism by which EGR3 could regulate expression of the *Bdnf* gene. Further studies will be necessary to determine whether EGR3 binds to these sites *in vivo*, and whether this binding may be functional.

**Table 1:** EGR3 binding sites identified in the *Bdnf* promoter using TFBIND.

ID^∗1^	Score^∗2^	Position^∗3^	Strand^∗4^	Consensus sequence^∗5^	Identified sequence^∗6^
V$EGR3_01	0.807500	1947	(-)	NTGCGTGGGCGK	CCTCCCACGTCA
V$EGR3_01	0.753542	2881	(-)	NTGCGTGGGCGK	ACTCGCACTCAT
V$EGR3_01	0.741042	3144	(-)	NTGCGTGGGCGK	CCGCATCCGCCT
V$EGR3_01	0.901667	3175	(-)	NTGCGTGGGCGK	ACGCCCGCGCAC
V$EGR3_01	0.748958	3179	(-)	NTGCGTGGGCGK	CCGCGCACACGC
V$EGR3_01	0.771042	3181	(-)	NTGCGTGGGCGK	GCGCACACGCGC
V$EGR3_01	0.805417	3187	(-)	NTGCGTGGGCGK	ACGCGCACACAC
V$EGR3_01	0.777083	3296	(-)	NTGCGTGGGCGK	CAGCCTGCGCAG

*^∗*1*^ID: transcription factor matrix ID (from TRANSFAC R.3.4). V: vertebrate. ^∗*2*^Score: degree of similarity between input sequence and registered sequence for the transcription factor binding sites at the position shown in the position column (ranging from 0 to 1, representing low–high degree of similarity). ^∗*3*^Position: location of putative EGR3 binding site in the 4000 nucleotides upstream of *Bdnf* transcription start site. ^∗*4*^Strand: location of EGR3 consensus sequences on + vs. - strand of DNA. ^∗*5*^Consensus sequence (fixed) of the transcription factor binding sites. K = G or T; N = any base pair. ^∗*6*^Putative EGR3 binding sequence identified by TFBIND.*

### Hippocampal Neuronal Numbers Are Not Affected by Developmental Absence of *Egr3*

**FIGURE 5:**
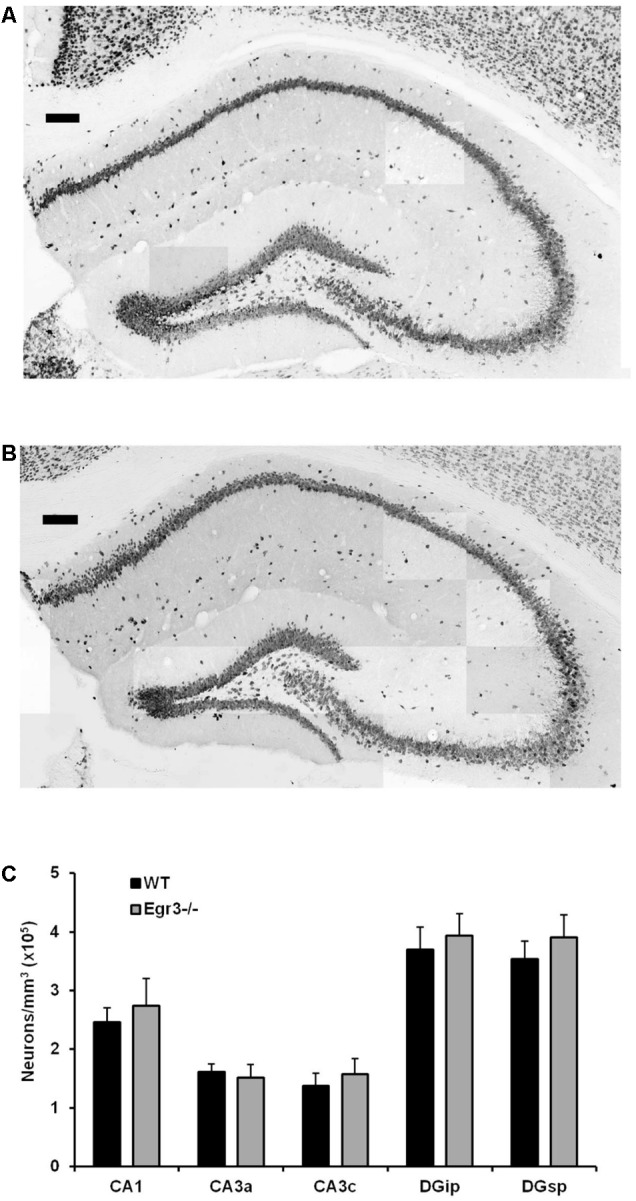
*Egr3* is not required for development of normal numbers of hippocampal neurons. Sample montaged photomicrographs from the hippocampus of anti-NeuN stained **(A)** WT and **(B)** *Egr3*–/– mice show no obvious signs of changes in gross morphology (scale bar = 100 μm). Consistent with this observation, **(C)** quantification of NeuN+ cells (*n* = 6 animals/genotype) demonstrated no significant differences in neuron density in any hippocampal region between WT (black) and *Egr3*–/– (gray) mice.

*Egr3*-/- mice lack expression of the functional gene throughout development and life. It is therefore possible that the absence of induction of *Bdnf* in the hippocampus following ECS in these animals may be a consequence of abnormal development, or survival, of hippocampal neurons in the absence of *Egr3*. To determine whether this may be the case, we conducted detailed regional cell counts of anti-NeuN antibody immunolabeled neurons in the hippocampus of matched pairs of adult *Egr3*-/- and WT littermate mice. WT and *Egr3*-/- brains display a comparable distribution and density of neurons (**Figure 5**). As expected (van Strien et al., 2009), a significant difference was observed on the basis of hippocampal region [*F*_(4,50)_ = 9.13, *p* < 0.001], with the DG showing the tightest cell packing density, while the CA3 regions were the least densely packed. However, there was no difference in the number of neurons in any hippocampal regions between *Egr3*-/- and WT control mice. No significant main effect of genotype [*F*_(1,50)_ = 1.19, *p* = 0.28] or interaction between genotype and hippocampal region [*F*_(4,50)_ = 0.10, *p* = 0.98] was observed.

### Dendritic Complexity Remains Normal in *Egr3*-/- Mice

**FIGURE 6:**
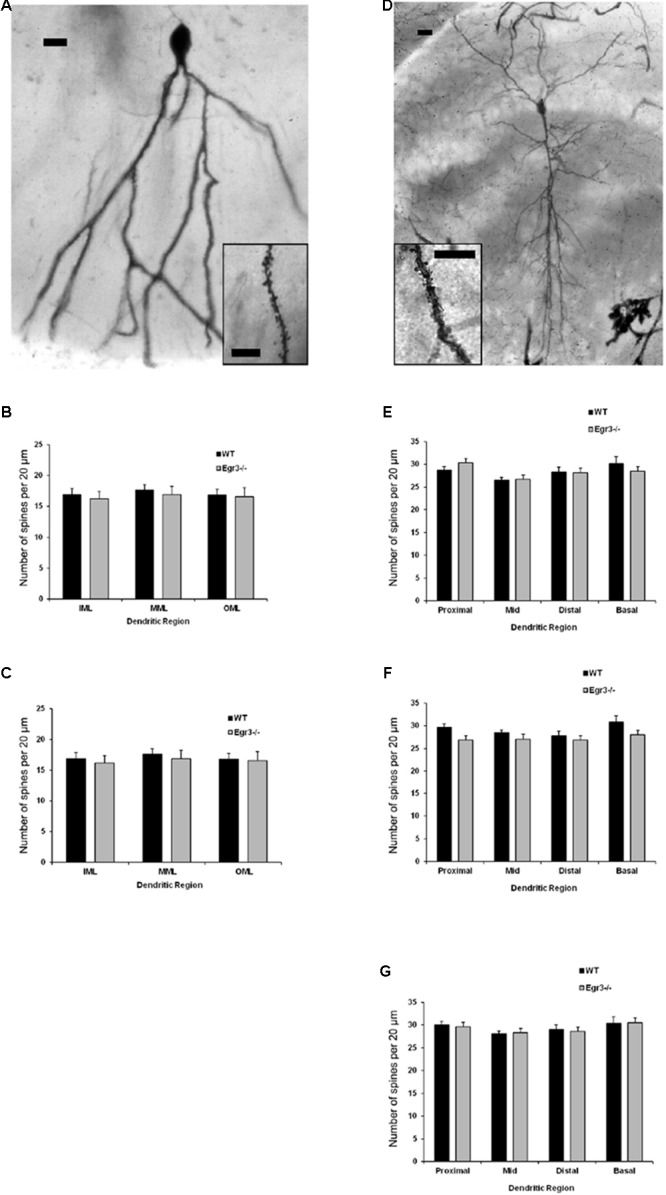
*Egr3* is not required for normal development of neuronal morphology. Samples of Golgi-Cox staining in **(A)** granule cells and **(D)** pyramidal cells shows impregnation of both gross dendritic structure (scale bar = 20 μm) as well as spines (inset, scale bar = 10 μm). Quantitative data show no statistically significant differences in either dendritic morphology or spine number between WT (black) and *Egr3*–/– (gray) mice in the **(B)** DG_sp_, **(C)** DG_ip_, **(E)** CA1, **(F)** CA3a/b, or **(G)** CA3c regions.

To further characterize how the absence of functional *Egr3* may alter the structure of hippocampal neurons, we conducted detailed analyses of cells impregnated with Golgi-Cox solution in adult WT and *Egr3*-/- littermate mice (**Figures 6A,B**). In pyramidal cells (**Table 2**), no significant differences were observed in the Sholl analysis [*F*_(1,24)_ = 2.71, *p* = 0.11] or branching order [*F*_(1,24)_ = 0.81, *p* = 0.38] on the basis of genotype. Moreover, no significant main effect of region was observed on Sholl analysis [*F*_(2,24)_ = 1.32, *p* = 0.29], or branching order [*F*_(2,24)_ = 0.68, *p* = 0.51], and no interaction between genotype and region was observed (*p* > 0.05 in all cases). Similarly, granule cells (**Table 3**) showed no significant differences in the Sholl analysis [*F*_(1,16)_ = 0.11, *p* = 0.75) or branching order [*F*_(1,16)_ = 1.678, *p* = 0.21] on the basis of genotype. A significant main effect of region was observed on Sholl analysis [*F*_(1,16)_ = 20.52, *p* < 0.001], indicating that granule cells in the DG_sp_ are significantly larger and more complex than granule cells from the DG_ip_, consistent with previous observations (Gallitano et al., 2016), although branching order was not significantly different [*F*_(1,16)_ = 3.15, *p* = 0.09]. No interaction between genotype and region was observed (*p* > 0.05 in all cases).

**Table 2:** Dendritic arborization in hippocampal cells of WT and *Egr3*-/- mice.

		WT						Egr3-/-					
		CA1		CA3a/b		CA3c		CA1		CA3a/b		CA3c	
		Apical	Basal	Apical	Basal	Apical	Basal	Apical	Basal	Apical	Basal	Apical	Basal
Branching	1	1.30 ± 0.21^1^	2.50 ± 0.47	2.53 ± 0.42	3.35 ± 1.07	1.77 ± 0.38	4.73 ± 0.85	1.13 ± 0.07	4.00 ± 0.10	2.80 ± 0.23	2.13 ± 0.22	2.01 ± 0.92	3.04 ± 0.17
Order	2	2.19 ± 0.55	4.75 ± 0.47	3.28 ± 0.27	3.50 ± 0.87	2.50 ± 0.84	4.38 ± 0.94	3.66 ± 0.63	6.10 ± 0.64	3.10 ± 0.17	2.50 ± 0.14	3.80 ± 1.04	3.46 ± 0.41
	3	5.89 ± 0.32	7.00 ± 0.74	3.05 ± 0.84	5.90 ± 0.87	2.53 ± 0.85	5.12 ± 0.78	6.08 ± 0.46	7.28 ± 0.88	4.00 ± 0.32	3.96 ± 0.17	3.20 ± 0.42	4.79 ± 0.22
	4	4.57 ± 0.39	7.00 ± 0.65	2.23 ± 0.31	5.50 ± 1.03	1.30 ± 0.78	5.30 ± 0.12	5.71 ± 0.34	5.23 ± 0.01	2.50 ± 0.27	3.38 ± 0.65	1.30 ± 0.16	2.83 ± 0.20
	5	6.02 ± 0.90	5.75 ± 1.08	3.03 ± 0.45	4.40 ± 1.03	2.37 ± 1.18	4.90 ± 0.10	4.30 ± 0.32	2.90 ± 0.06	1.80 ± 0.10	1.13 ± 0.07	2.00 ± 0.32	0.67 ± 0.19
	6	2.00 ± 0.49	2.50 ± 0.95	1.70 ± 0.37	2.10 ± 0.07	0.83 ± 0.59	1.80 ± 0.09	2.17 ± 0.31	1.15 ± 0.38	1.40 ± 0.23	0.50 ± 0.14	0.60 ± 0.12	0.29 ± 0.02
	7	1.57 ± 0.31	1.00 ± 0.47	1.60 ± 0.92		0.27 ± 0.19		0.89 ± 0.23	0.01 ± 0.01	4.00 ± 0.32		0.51 ± 0.43	
	8+	0.84 ± 0.27	0.25 ± 0.12	0.50 ± 0.29		–		0.57 ± 0.33	–	–		–	
	Total	24.51 ± 2.48	30.75 ± 4.90	19.90 ± 3.68	16.48 ± 2.73	16.17 ± 3.65	16.80 ± 2.77	22.94 ± 1.54	25.19 ± 4.98	21.60 ± 3.12	15.93 ± 3.34	14.95 ± 1.99	13.10 ± 0.64

Scholl (μm)	20	2.03 ± 0.14	1.50 ± 0.70	1.70 ± 0.17	6.78 ± 0.56	1.73 ± 0.30	6.93 ± 0.19	1.91 ± 0.20	7.48 ± 1.00	1.70 ± 0.17	5.80 ± 0.46	1.38 ± 0.33	5.78 ± 0.01
	40	3.50 ± 0.12	3.75 ± 0.60	2.58 ± 0.68	8.78 ± 1.14	3.68 ± 0.62	9.73 ± 0.30	3.58 ± 0.17	10.88 ± 0.65	2.58 ± 0.68	6.80 ± 0.46	2.75 ± 0.72	6.95 ± 0.84
	60	5.19 ± 0.22	5.00 ± 2.14	3.63 ± 0.36	7.15 ± 1.36	5.28 ± 0.27	10.43 ± 0.48	5.04 ± 0.31	10.03 ± 0.45	3.63 ± 0.36	5.58 ± 0.48	3.25 ± 0.82	5.25 ± 1.01
	80	4.51 ± 0.26	4.75 ± 1.72	4.85 ± 0.38	4.63 ± 2.09	6.60 ± 0.35	6.68 ± 1.20	5.61 ± 0.06	8.53 ± 0.74	4.85 ± 0.38	3.78 ± 0.59	5.25 ± 1.43	3.83 ± 0.91
	100	4.22 ± 0.57	6.00 ± 3.51	4.98 ± 0.45	1.85 ± 0.95	6.45 ± 1.13	5.45 ± 1.18	5.79 ± 0.53	4.00 ± 1.15	4.98 ± 0.45	1.73 ± 0.27	5.34 ± 1.24	2.75 ± 0.72
	120	5.18 ± 0.32	7.00 ± 3.79	4.65 ± 0.66	0.85 ± 0.38	4.35 ± 0.49	3.15 ± 0.78	5.78 ± 0.38	2.00 ± 0.58	4.65 ± 0.66	0.95 ± 0.26	6.13 ± 1.47	0.65 ± 0.09
	140	4.55 ± 0.45	4.25 ± 2.04	5.08 ± 0.76	0.50 ± 0.29	3.48 ± 0.16	1.35 ± 0.66	5.27 ± 0.59	0.85 ± 0.20	5.08 ± 0.76	0.20 ± 0.12	6.75 ± 1.67	0.19 ± 0.10
	160	5.63 ± 0.60	4.75 ± 1.98	4.48 ± 1.00	–	3.38 ± 0.36	–	5.08 ± 0.70	0.50 ± 0.15	4.48 ± 1.00	–	6.50 ± 1.90	–
	180	6.00 ± 0.53	4.50 ± 0.68	3.88 ± 1.23	–	2.50 ± 0.29	–	4.88 ± 0.65	0.19 ± 0.06	3.88 ± 1.23	–	4.13 ± 1.30	–
	200	5.09 ± 0.41	3.50 ± 0.24	3.00 ± 1.15	–	1.68 ± 0.53	–	4.73 ± 0.57	0.10 ± 0.06	3.00 ± 1.15	–	3.00 ± 0.94	–
	220	3.50 ± 0.41	2.75 ± 0.24	2.08 ± 0.76	–	1.35 ± 0.49	–	4.46 ± 0.48	0.03 ± 0.09	2.08 ± 0.76	–	1.50 ± 0.47	–
	240	1.99 ± 0.21	1.75 ± 0.98	1.90 ± 0.52	–	1.15 ± 0.38	–	2.32 ± 0.10	–	1.90 ± 0.52	–	1.00 ± 0.26	–
	260	1.11 ± 0.10	0.50 ± 0.28	1.28 ± 0.30	–	0.40 ± 0.23	–	1.40 ± 0.02	–	1.28 ± 0.30	–	0.50 ± 0.16	–
	280	0.69 ± 0.18	–^1^	–	–	0.20 ± 0.12	–	0.58 ± 0.17	–	–	–	0.13 ± 0.06	–
	300	0.30 ± 0.06	–	–	–	–	–	0.13 ± 0.07	–	–	–	–	–
	320	0.11 ± 0.09	–	–	–	–	–	–	–	–	–	–	–
	Total	52.00 ± 9.93	39.10 ± 19.02	44.95 ± 4.88	26.30 ± 4.33	42.60 ± 4.23	33.08 ± 4.93	52.63 ± 8.30	35.25 ± 6.33	44.95 ± 4.88	24.83 ± 3.64	42.15 ± 3.94	35.20 ± 4.58

*^*1*^All data are reported as mean ±*SE*. ^*2*^Denotes that no observations could be made in this category.*

**Table 3:** Dendritic arborization in granule cells of WT and *Egr3*-/- mice.

		WT		Egr3-/-	
		DG_sp_	DG_ip_	DG_sp_	DG_ip_
Branching	1	2.05 ± 0.17^1^	2.04 ± 0.10	2.23 ± 0.12	2.08 ± 0.50
Order	2	4.13 ± 0.36	3.89 ± 0.28	4.65 ± 0.22	3.93 ± 0.47
	3	5.08 ± 0.39	4.91 ± 0.21	5.55 ± 0.27	5.03 ± 0.68
	4	3.63 ± 0.16	3.58 ± 0.77	4.38 ± 0.35	4.28 ± 1.02
	5	1.225 ± 0.25	1.70 ± 0.39	1.50 ± 0.36	1.55 ± 1.15
	6	0.45 ± 0.17	0.40 ± 0.21	0.63 ± 0.31	0.35 ± 0.37
	7	0.45 ± 0.16	0.40 ± 0.21	0.53 ± 0.26	0.30 ± 0.37
	8+	0.09 ± 0.04	0.01 ± 0.01	0.10 ± 0.06	0.05 ± 0.01
	Total	17.00 ± 0.61	16.91 ± 1.20	19.55 ± 1.36	17.55 ± 2.74

Scholl (μm)	20	2.85 ± 0.28	3.13 ± 0.85	3.08 ± 0.26	3.27 ± 0.28
	40	4.425 ± 0.20	4.48 ± 1.20	4.55 ± 0.38	4.39 ± 0.43
	60	4.7 ± 0.16	5.83 ± 0.73	5.58 ± 0.49	5.53 ± 0.45
	80	5.73 ± 0.20	6.05 ± 0.71	6.32 ± 0.38	5.94 ± 0.35
	100	6.10 ± 0.16	6.33 ± 0.84	6.80 ± 0.39	6.02 ± 0.58
	120	6.85 ± 0.19	6.63 ± 0.62	7.20 ± 0.31	6.52 ± 0.48
	140	6.975 ± 0.21	6.50 ± 0.76	7.75 ± 0.49	6.55 ± 0.38
	160	7.05 ± 0.26	6.30 ± 0.85	7.90 ± 0.40	6.50 ± 0.41
	180	6.98 ± 0.28	5.65 ± 1.20	7.85 ± 0.46	5.95 ± 0.38
	200	6.58 ± 0.27	5.08 ± 0.93	7.48 ± 0.50	5.45 ± 0.49
	220	6.53 ± 0.23	4.48 ± 0.96	6.83 ± 0.50	5.40 ± 0.49
	240	6.10 ± 0.18	3.48 ± 1.34	6.43 ± 0.44	4.36 ± 0.45
	260	5.28 ± 0.25	2.50 ± 1.45	5.95 ± 0.31	3.55 ± 0.50
	280	4.65 ± 0.33	1.70 ± 1.80	5.15 ± 0.28	2.29 ± 0.37
	300	3.5 ± 0.27	0.95 ± 1.83	3.80 ± 0.23	1.37 ± 0.38
	320	2.3 ± 0.20	0.40 ± 0.97	2.48 ± 0.29	0.64 ± 0.29
	340	1.5 ± 0.44	0.25 ± 0.96	1.88 ± 0.55	0.48 ± 0.19
	Total	88.08 ± 2.15	69.68 ± 10.45	97.01 ± 4.26	74.17 ± 5.41

*^*1*^All data are reported as mean ±*SE*.*

### Spine Density Is Not Affected by Loss of *Egr3*

Consistent with the dendritic analysis, ANOVAs showed no significant differences in spine density in any brain region (**Figure 6**). In pyramidal cells, no significant difference on the basis of genotype in the density of basal spines in stratum oriens [*F*_(1,24)_ = 0.35, *p* = 0.5588], in apical spines in stratum radiatum proximal [*F*_(1,24)_ = 0.64, *p* = 0.43] or distal [*F*_(1,24)_ = 0.35, *p* = 0.56] to the soma, nor in stratum lacunosum/moleculare [*F*_(1,24)_ = 1.31, *p* = 0.26]. Similarly, no significant differences were observed on the basis of hippocampal region (i.e., CA1, CA3a/b, and CA3c) basal spines in stratum oriens [*F*_(2,24)_ = 0.92, *p* = 0.41], in stratum radiatum proximal [*F*_(2,24)_ = 0.42, *p* = 0.66] or distal [*F*_(2,24)_ = 0.27, *p* = 0.77] to the soma, nor in stratum lacunosum/moleculare [*F*_(2,24)_ = 0.5624, *p* = 0.5772]. Similarly, no significant interactions were observed between region and genotype (*p* > 0.05 in all cases).

In granule cells, there was no significant effect of genotype on the density of spines in the IML [*F*_(1,16)_ = 0.31, *p* = 0.58], MML [*F*_(1,16)_ = 0.14, *p* = 0.71], or OML [*F*_(1,16)_ = 0.33, *p* = 0.58]. Similarly, no significant differences in spine density were observed on the basis of blade of the DG (i.e., DG_sp_ vs. DG_ip_) in IML [*F*_(1,16)_ = 0.28, *p* = 0.64], MML [*F*_(1,16)_ = 0.33, *p* = 0.57], or OML [*F*_(1,16)_ = 0.76, *p* = 0.30]. Similarly, no significant interactions were observed between region and genotype (*p* > 0.05 in all cases).

## Discussion

*Egr3* is an IEG transcription factor that is induced in response to environmental stimuli (Thompson et al., 2010; Maple et al., 2015). We have hypothesized that dysfunction of *Egr3* could disrupt the brain’s normal neurobiological response to stress, resulting in increased risk to develop mental illnesses such as schizophrenia and bipolar disorder (Gallitano-Mendel et al., 2007, 2008; Williams et al., 2012; Huentelman et al., 2015; Pfaffenseller et al., 2016; Marballi and Gallitano, 2018). In support of this hypothesis, our prior work has shown that *Egr3*-/- mice display behavioral abnormalities consistent with animal models of psychotic disorders that can be reversed by administration of medications used to treat these illnesses in humans (Gallitano-Mendel et al., 2007, 2008). In addition, *Egr3* has been associated with risk for schizophrenia in Japanese, Korean, Han Chinese populations, and populations of European Descent (Yamada et al., 2007; Kim et al., 2010; Zhang et al., 2012; Huentelman et al., 2015), and *Egr3* expression is decreased in the brains of patients with schizophrenia, compared with controls (Mexal et al., 2005; Yamada et al., 2007). Bioinformatics approaches have identified *Egr3* as a central gene in a network of transcription factors and microRNAs implicated in schizophrenia risk (Guo et al., 2010), as well as a master regulator of genes differentially expressed in the brains of bipolar disorder patients in two independent cohorts (Pfaffenseller et al., 2016). Together, these findings suggest that disruption of *Egr3* activity, or function of other proteins that act either upstream or downstream in the EGR3 pathway, may mediate both the genetic and environmental factors that contribute to risk for severe mental illnesses (Marballi and Gallitano, 2018).

Since EGR3 functions as a transcription factor, we hypothesized that the downstream target genes regulated by EGR3 may also influence risk for psychotic disorders. IEGs, including *Egr3*, are expressed at low levels at baseline. We therefore conducted ECS to induce rapid, high-level, expression of *Egr3* (O’Donovan et al., 1998). We used a microarray approach to compare genes expressed 1 h following ECS in the hippocampus of WT, compared to *Egr3*-/-, mice to identify EGR3-dependent target genes. This study revealed increased expression of *Bdnf*, in the WT mice, but not in the *Egr3*-/- mice, indicating that ECS-induced *Bdnf* expression requires *Egr3*.

BDNF is a neurotrophic factor that promotes growth and differentiation of neurons and synapses (Barde et al., 1987; Binder and Scharfman, 2004; Bennett and Lagopoulos, 2014). In addition, BDNF has been found to play a role in the therapeutic effectiveness of treatments for psychiatric illnesses. Animals treated with antidepressant medications demonstrate increased expression of *Bdnf* in the hippocampus (Bjorkholm and Monteggia, 2016). Disruption of BDNF function is implicated in the pathology underlying numerous psychiatric disorders, ranging from schizophrenia and bipolar disorder to depression (Grande et al., 2010; Lee and Kim, 2010; Autry and Monteggia, 2012). Reduction of hippocampal BDNF attenuates the effect of antidepressants (Monteggia et al., 2004; Adachi et al., 2008), and infusion of BDNF into the hippocampus reverses depression-like behavior in rodents (Shirayama et al., 2002).

The mouse *Bdnf* gene consists of nine exons; eight of which are in the 5′ untranslated region of the gene (exons I–VIII) and only one of which encodes protein (exon IX; Aid et al., 2007). Different *Bdnf* splice variants are formed from a combination of one of the 5′ untranslated exons (I–VIII) with the common protein-coding exon (exon IX; Liu et al., 2005; McDowell et al., 2010). Two alternative polyadenylation signals result in transcription termination and result in either the short (0.3 kB) or long (2.9 kB) 3′ UTR of *Bdnf* (Timmusk et al., 1993).

Prior studies have shown that activity-dependent transcription of *Bdnf* can be mediated by calcium influx (Shieh and Ghosh, 1999; West et al., 2001; Aid et al., 2007; Hong et al., 2008; West, 2008). Calcium responsive elements, including cAMP-responsive element-binding (CREB) protein (West et al., 2001), Carf (Tao et al., 2002), calcium-dependent phosphorylation of the methyl CpG binding protein 2 (MeCP2), and the basic helix-loop-helix upstream signaling factors (USFs; Chen et al., 2003), positively regulate expression of *Bdnf*. In contrast, stressful environmental stimuli, ischemia (Lindvall et al., 1992), and prenatal stress (Boersma et al., 2014) lead to decreased expression levels of *Bdnf* in the hippocampus.

One of the stimuli known to activate *Bdnf* is ECS. ECS is the experimental equivalent of ECT, a treatment that has been in use for more than 80 years, and that remains the most effective treatment for severe mood disorders and psychotic disorders with a mood component (Fink, 2001, 2014; Pagnin et al., 2004; Rosenquist et al., 2014). ECT results in increased levels of BDNF in peripheral blood in humans (Bocchio-Chiavetto et al., 2006). Studies in rodents demonstrate that ECS induces high levels of BDNF expression in the hippocampus (Altar et al., 2004). ECS also induces expression of IEGs, including *Egr3*, as well as other growth factors in addition to BDNF, and stimulates neurogenesis and dendritic sprouting in the hippocampus (Duman and Vaidya, 1998; Malberg et al., 2000; Altar et al., 2004; Perera et al., 2007; Kato, 2009; Youssef and McCall, 2014; Thomann et al., 2017).

Our findings show that hippocampal induction of *Bdnf* in response to ECS requires *Egr3*. The presence of EGR3 binding sites in the *Bdnf* promoter suggests the possibility that EGR3, a transcription factor, may directly regulate expression of *Bdnf*. However, since the current study did not confirm binding of EGR3 to these sites *in vivo*, we are not able to determine this at this time.

Exon IV in particular seems to be an important component of activity-dependent *Bdnf* expression, as deletion of exon IV impedes both ECS-induced, and sleep deprivation-mediated, activation of several *Bdnf* transcripts *in vivo* (Martinowich et al., 2011). Notably, both ECS and sleep deprivation activate expression of *Egr3* as well (Thompson et al., 2010; Maple et al., 2015). Deletion of exon IV also causes impairments in spatial memory reversal and fear memory extinction (Sakata et al., 2013), deficits in GABAergic transmission in the cortex (Sakata et al., 2009), and impairment of hippocampal synaptic plasticity (Sakata et al., 2013) *in vivo*.

Another important activity-induced exon in the hippocampus is exon VI (Chiaruttini et al., 2008). Both *Bdnf* exon IV and VI contribute significantly to BDNF protein levels in the hippocampus (Maynard et al., 2016). We found that both exon IV and VI expression failed to show induction in *Egr3*-/- mice following ECS compared to WT mice. Follow-up bioinformatic analysis of the 4 kb proximal upstream region of *Bdnf* using TFBind revealed eight high-probability EGR3 binding sites (**Table 1**). Further studies will be important to confirm the binding of EGR3 to these sites and confirm the specific conditions and cell types in which they occur.

Psychotic disorders are characterized by cognitive impairments (Goff et al., 2011). In animals, disrupting the expression of BDNF results in deficits in learning, memory, attention, executive function, and cognition (Sharma and Antonova, 2003). Both *Egr3* and BDNF play critical roles in hippocampal synaptic plasticity, a process associated with memory formation. Specifically, long-term depression, a form of hippocampal plasticity that is facilitated by novelty and stress exposure, requires *Egr3*, and is stimulated by the immature form of BDNF, proBDNF (Kim et al., 1996; Woo et al., 2005; Gallitano-Mendel et al., 2007; Yang et al., 2014; Bukalo et al., 2016). The results of these, and many other studies, have led to the hypothesis that BDNF may be a critical molecule in mediating the therapeutic effects of psychiatric therapies, including ECT (Bocchio-Chiavetto et al., 2006; Li et al., 2016).

One limitation of our study is that it employed mice that lack *Egr3* function throughout development and life. It is therefore possible that the failure of ECS to induce *Bdnf* expression in *Egr3*-/- mice could be explained by a developmental deficit of the cells that normally express *Bdnf*. However, our neuroanatomical studies demonstrated no regional differences in neuronal counts in the DG or CA regions of the hippocampus in *Egr3*-/- mice. Furthermore, no differences were found in the spine density or branch order of pyramidal cells, or in the density DG granule cells.

The absence of neuroanatomical deficits in *Egr3*-/- mice is supported by our prior findings of normal activation of *Arc* expression in DG granule cells following exposure to a novel environment (Maple et al., 2017) and prior studies demonstrating seizure-induced *Arc* expression in the hippocampus of *Egr3*-/- mice (Li et al., 2005). However, this *Arc* expression dissipates over several hours in *Egr3*-/- DG cells, compared to WT mice in which *Arc* expression perdures for up to 8 h (Maple et al., 2017). Thus, the hippocampal DG granule cells of *Egr3*-/- mice are capable of expressing *Arc* in response to activity, but maintenance of this expression requires *Egr3*. Our results indicate that the numbers of DG cells remain consistent in the WT and *Egr3*-/- mice, which suggests that the loss of *Bdnf* in these cells is due to failed activation in the absence of *Egr3*.

Another limitation is that, although we show that *Bdnf* fails to be induced following ECS in the *Egr3*-/- mice, and the *Bdnf* promoter contains putative EGR3 binding sites, we cannot rule out the possibility that an intermediary factor regulates expression of *Bdnf*. Future studies will employ chromatin immunoprecipitation (ChIP) to assess binding of EGR3 to the *Bdnf* promoter in the hippocampus, as well as luciferase reporter assays to demonstrate functionality of this binding *in vitro*.

The expression of IEGs is, by definition, independent of protein synthesis. Since exons III and IV of the mouse BDNF have been reported to have IEG activity (Lauterborn et al., 1996; Binder and Scharfman, 2004; Sakata et al., 2009), this indicates that *Bdnf* transcription should be dependent upon transcription factor proteins that are already present and poised for activation. However, in our study, we demonstrate that *Egr3*, an IEG transcription factor, mediates *Bdnf* mRNA expression in response to ECS. Specifically, we found that *Egr3* is required for ECS-induced expression of *Bdnf* exons IV and VI, with a larger effect on exon IV, in the hippocampus. Since our study examined gene expression 1 h following ECS, it is possible that *Bdnf* is initially activated in the brains of *Egr3*-/- mice, but fails to be maintained in the absence of *Egr3*, as seen with *Arc* expression in prior studies by our group and others (Li et al., 2005; Maple et al., 2017). If this is the case, then the induced IEG *Bdnf* transcripts would have to be degraded within 1 h following ECS, a timeframe more rapid than that of *Arc*.

Our studies identify the novel requirement of EGR3 for hippocampal *Bdnf* expression in response to ECS. We further show that EGR3 is required for seizure-induced expression of *Bdnf* transcripts containing exons IV and VI. Notably, these two isoforms are the greatest contributors to total BDNF protein in the hippocampus (Maynard et al., 2016). These data suggest the possibility that EGR3 may play a role in other forms of activity-dependent *Bdnf* induction in the hippocampus, such as that induced by high-frequency stimulation or exposure to novelty. However, this will have to be tested in future studies. This finding is of interest as both *Egr3* and *Bdnf* share several common features. These include essential roles in hippocampal long-term depression (Gallitano-Mendel et al., 2007; Novkovic et al., 2015; Bukalo et al., 2016) calcium-signaling-dependent activation (Mittelstadt and Ashwell, 1998; Shieh et al., 1998), and genetic association with schizophrenia (Gratacos et al., 2007; Yamada et al., 2007; Kim et al., 2010; Ning et al., 2012; Zhang et al., 2012; Huentelman et al., 2015). We have hypothesized that both *Bdnf* and *Egr3* are crucial components of a biological pathway implicated in mental illness risk (Marballi and Gallitano, 2018). It will be interesting to determine whether activity-dependent, EGR3-mediated *BDNF* expression contributes to the therapeutic effects of ECT on the mood and psychotic symptoms of patients with severe psychiatric illnesses.

## Author Contributions

MC accumulated images and data for the manuscript and assisted in analyzing NeuN counts. BR contributed to data analysis and image collection for the hippocampal neuroanatomical studies. SB analyzed the original microarray study, identified downstream targets of the immediate early gene, *Egr3*, and developed figures and tables for the binding regions of EGR3 within the Bdnf promoter. KKM validated the work of SB, performed validation studies in the female cohorts of mice, analyzed the qRT-PCR results, formulated graphs, and assisted in the conceptualization of the experiments and the development of the manuscript. AG funded the research with her NIMH funds, conceptualized the project, and developed the hypothesis of immediate early genes synergistically acting with genetic factors to contribute toward the pathogenesis of schizophrenia. DM utilized his funds obtained from the Natural Sciences and Engineering Research Council of Canada, collaborated with AG to establish the theory of regulation of *Bdnf* by *Egr3*, and contributed directly to the manuscript with his writing, editing, and neuroanatomical studies. KTM performed ECS, collected tissue, performed *in situ* hybridization, analyzed the results, and assisted in writing the manuscript.

## Conflict of Interest Statement

The authors declare that the research was conducted in the absence of any commercial or financial relationships that could be construed as a potential conflict of interest.

## Footnotes

1 http://tfbind.hgc.jp

2 http://genome.ucsc.edu/
